# Responses to Success: Seeking Pleasant Experiences before a Task Is Complete?

**DOI:** 10.1371/journal.pone.0135952

**Published:** 2015-08-21

**Authors:** Marina Schall, Thomas Goetz, Sarah E. Martiny, Rebecca Maymon

**Affiliations:** 1 Department of Empirical Educational Research, University of Konstanz, Konstanz, Germany; 2 Thurgau University of Teacher Education, Kreuzlingen, Switzerland; 3 Department of Psychology, UiT The Arctic University of Norway, Tromsø, Norway; 4 Department of Educational and Counselling Psychology, McGill University, Montreal, Canada; Centre national de la recherche scientifique, FRANCE

## Abstract

Although engaging in pleasant experiences following successful performance may be hedonically rewarding, in the present research we proposed that individuals might forego pleasant experiences when they have not yet completed a task. In Study 1 (*N* = 100), participants reported the extent to which they would like to engage in pleasant experiences in a hypothetical situation where their performance outcome on a task (successful vs. average) and task completion (task in progress vs. completed) were manipulated. In Study 2 (*N* = 115), participants were in a real situation in which they achieved either a successful or average performance outcome. Task completion was manipulated (task in progress vs. completed) and motivation to engage in a pleasant experience was assessed by a behavioral measure. Results of both studies provided support for our prediction by showing individuals to have a lower desire to engage in pleasant experiences following successful, but not average, performance when the task was in progress than when it was complete. These findings are discussed in light of the underlying mechanisms and consequences of the tendency to forego pleasant experiences.

## Introduction

The potential benefits and costs of seeking pleasure in life have been a longstanding philosophical dispute. Pleasure is defined by a “positive experienced state that we seek and that we try to maintain or enhance” [1]. Individuals seek pleasant experiences due to their hedonic motivation, as pleasant experiences feel good per definition [2, 3]. In the present research, we investigated individuals’ seeking of pleasant experiences specifically following successful achievements.

In particular, after achieving successful performance, individuals may perceive indulging in pleasant experiences as rewarding because it prolongs their positive emotions following success. Indeed, studies suggest that individuals typically strive to protect their positive emotional states [4] and have shown that in positive moods individuals prefer to engage in pleasant activities more than in neutral moods [5, 6]. Moreover, engaging in pleasant experiences following success can also reflect individuals’ desire to savor their outstanding performance [7, 8]. This motivation can be evident in everyday life, such as when individuals gratify themselves following success, share positive events with others, and celebrate such events [9–11]. Thus, previous studies imply that engaging in pleasant experiences following successful achievements may help individuals to satisfy their hedonic needs and to enjoy their achievements. In the present research, we further proposed that whether individuals will seek pleasant experiences following successful performance or not may depend on the situation in which individuals have achieved their success.

Indeed, existing research shows that engaging in pleasant experiences not only feels good but is also associated with pursuing short-term goals by way of giving into short-term desires and temptations. This research has found that indulging in pleasant emotions can interfere with the pursuit of one’s long-term goals, such as focusing on an ongoing performance [12]. This finding is in line with research stating that the experience of pleasure can evoke openness for other possibilities rather than the task or goal currently being pursued and signal that no effort is needed as one’s goal has been achieved [13–16]. Thus, we assumed that in situations where individuals have not yet completed a task and their next performance lies ahead, engaging in pleasant experiences following successful performance might be viewed as less beneficial. Rather than striving for pleasant experiences individuals may instead strive to stay focused on the incomplete task or, in other words, “forego” pleasant experiences for the sake of future positive outcomes [17–19]. However, when the task has been finished, engaging in pleasant experiences following one’s successful performance may correctly indicate that one’s goal has been successfully reached.

As such, we hypothesized that seeking pleasant experiences may be a less favored response to successful performance when the task is in progress, as compared to when the task is completed. To date, such a prediction has yet to be investigated as previous research has drawn little attention to how individuals respond to successful performances and has primarily investigated how individuals deal with setbacks [10].

## The Present Research

In the present research, we investigated if the extent to which individuals seek pleasant experiences following successful performance depends on the performance situation. We predicted that individuals would be less motivated to engage in pleasant experiences following success when the task was in progress than when the task was complete.

Moreover, we aimed to ensure that the effect of the performance situation (i.e., task completion) on the desire for pleasant experiences was indeed specific to successful performance (and did not reflect an overall effect on the experience of tension or relief). Thus, in addition to individuals who attained successful performance, we implemented a control group who attained average performance. Because average performance is typically experienced less positively and as less rewarding than success, we did not expect individuals to differ in their motivation to seek rewarding or pleasant experiences following average performance depending on whether the task was complete or in progress. Of course, below average performance is also less positively experienced compared to successful performance. However, the use of a control group with average rather than below average performance attainment was perceived as more suitable to test our hypothesis because of potential floor effects. Moreover, we could not rule out that individuals may also seek pleasant experiences following performance failure in order to overcome negative emotions.

We tested the hypothesis that individuals will seek pleasant experiences following success, but not average performance, less when the task is still in progress than when complete in two experimental studies. In Study 1, participants were provided a written scenario depicting a hypothetical situation in which participants attained either successful or average performance on a task which was either in progress or completed. Seeking pleasant experiences was assessed by a self-report measure. In Study 2, participants were in a real situation in which they achieved either successful or average performance on a task which was either in progress or completed. We used a behavioral assessment of motivation to seek pleasant experiences. As previous research has found that individual preferences for pleasant experiences may be influenced by personality traits (i.e., self-esteem, extraversion [20]), in both studies, we additionally aimed to control for potential effects of these personality variables.

## Ethics Statement

The procedure of the studies was in compliance with the Ethical Principle of the WMA Declaration of Helsinki and deemed appropriate by the Institutional Review Board of the University of Konstanz. Participation was voluntary and only those students who signed a consent form participated in the study, in compliance with the ethical code of conduct of the American Psychological Association [21] and the ethic principles of the laboratory of the Economic Institute at the University of Konstanz. Students were recruited anonymously [22], and the questionnaires and data files contained only anonymous data. Unnecessary deception of participants was avoided. Participants were thoroughly debriefed and financially rewarded for their participation.

## Study 1

In Study 1, we tested the prediction that individuals would seek pleasant experiences following successful performance, but not average performance, less when the task was still in progress than when complete. To do so, we used a scenario in which participants were provided with a hypothetical situation. In that situation, both the outcome on a task (successful vs. average) and task completion (task in progress vs. completed) were manipulated. Participants were then asked to indicate the extent to which they would like to engage in pleasant experiences.

### Method

#### Participants and Design

One hundred German university students participated in the experiment (*M*_age_ = 21.03, *SD*_age_ = 2.42; 69 females) and were randomly assigned to one of four experimental conditions. We used a between-participants design to evaluate the effect of performance outcome (successful vs. average) and completion of the task (task in progress vs. completed) on the extent of pleasant experience seeking.

#### Procedure

The study was conducted at the university before a regular lecture and was introduced as a study on students’ learning behavior. Participants were informed about the confidentiality of their responses and were provided with a six-page questionnaire. The questionnaires were identical for all participants with the exception of the scenario situation.

We chose a situation that was considered typical of university students’ lives. Participants were asked to imagine that they are taking their exams at the end of the semester and have just passed an oral exam. In the successful outcome condition, participants read that they had answered all questions during the exam correctly and received a very good grade. In the average outcome condition, participants read that they had not answered all questions correctly and thus had received an average grade. To manipulate task completion, participants read that the exams have not been completed and another exam lay ahead of them (task in progress) or that they completed their exams (task completed).

Participants were asked to imagine the situation and to indicate the extent to which they would like to engage in pleasant experiences after passing the oral exam. Participants were asked how they would feel about such a (successful vs. average) result in an examination and how well they could imagine the situation. Then, they answered items assessing self-esteem and extraversion, as well as demographic items, were thanked and provided a chocolate bar in exchange for their participation.

#### Measures. Pleasant experience seeking

To assess pleasant experience seeking participants were asked how much they would like “to engage in pleasant experiences,” “to do something which makes [them] feel happy,” and “to do something which makes [them] feel pleasure” in that particular situation on a 7-point scale (1 = *not at all*; 7 = *very much*; α = .84).

#### Emotional valence of the outcome

To evaluate the emotional valence of the performance outcome participants were asked how they would feel about a (successful vs. average) result in an exam and to report their feelings on the Affect Evaluation Scale [23] ranging from 1 = *negative feelings* to 7 = *positive feelings*.

#### Self-esteem and extraversion

We used a translated Rosenberg’s [24] 10-item Self-Esteem Scale and a 12-item extraversion scale from the German version of Costa and McCrae’s [25] Big Five Personality Inventory [26]. Participants were asked to indicate the extent to which they disagree or agree with each of the statements on a 7-point scale (1 = *strongly disagree*; 7 = *strongly agree*; α = .87 for self-esteem, α = .77 for extraversion).

### Results

#### Positive Valence of the Outcome

We conducted a univariate analysis of variance on the emotional valence of the performance outcome with outcome and task completion as between-participants factors. Results showed a significant main effect of outcome, *F*(1, 96) = 53.76, *p* < .001, η_p_^2^ = .61. As expected, a successful outcome was evaluated overall more positively (*M* = 6.31, *SD* = 1.09) than an average performance outcome (*M* = 3.49, *SD* = 1.23). The effects of task completion and the interaction between task completion and outcome on the emotional valence were not significant, *F*s ≤ 0.59, *p*s ≥ .12, η_p_^2^ ≤ .02.

#### Pleasant experience seeking

**Fig 1:**
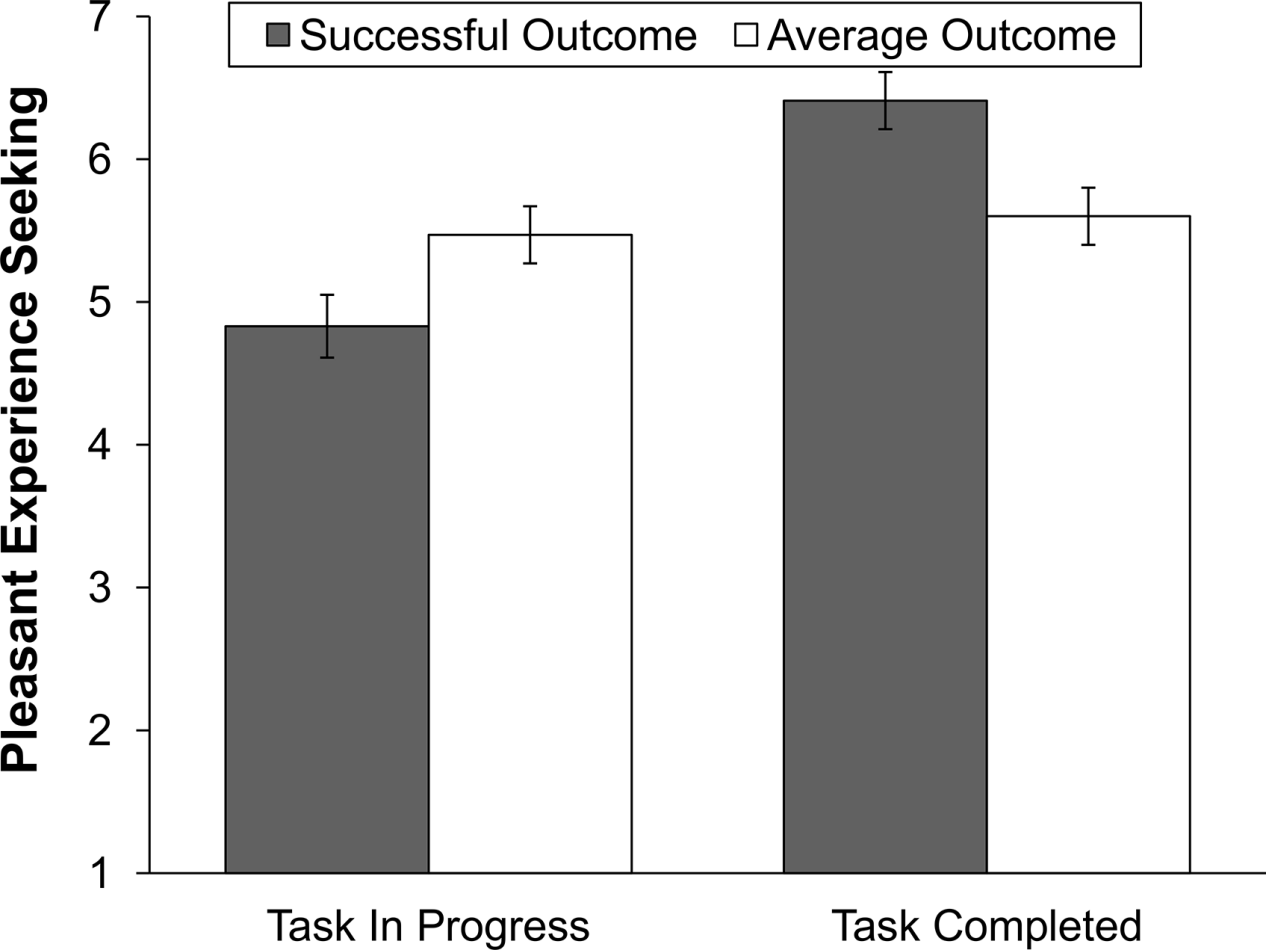
Group means and standard errors for motivation to seek pleasant experiences after a successful versus an average outcome in both the task in progress and task completed conditions.

We conducted a univariate analysis of variance on motivation to seek pleasant experiences with performance outcome and task completion as between-participants factors, and self-esteem and extraversion as covariates. Results showed a non-significant effect of performance outcome, *F*(1, 91) = 0.16, *p* = .69, η_p_^2^ = .002, and a significant effect of task completion, *F*(1, 91) = 16.61, *p* < .001, η_p_^2^ = .15. This significant effect was qualified by a significant interaction between outcome and task completion, *F*(1, 91) = 11.71, *p* = .001, η_p_^2^ = .11. We conducted post-hoc tests to investigate the differences in motivation to engage in pleasant experiences across the conditions in detail. Means and standard errors are displayed in Fig 1. In support of our hypothesis, results of post-hoc tests revealed that participants reported significantly lower motivation to seek pleasant experiences following successful performance when the task was in progress (*M* = 4.83, *SE* = .22) than when the task was completed (*M* = 6.41, *SE* = .20), *p* < .001, *d* = 1.53. No significant difference in motivation was found following average performance across the task completion conditions, *p* = .66, *d* = 0.13, indicating that the effect of task completion was specific to successful individuals.

Moreover, results further showed that motivation to seek pleasant experiences was significantly higher following successful performance (*M* = 6.41, *SE* = .20) than average performance (*M* = 5.60, *SE* = .22) in the task completed condition, *p* = .008, *d* = 0.78. However, in the task progress condition, motivation to seek pleasant experiences was significantly lower following successful performance (*M* = 4.83, *SE* = .22) than average performance (*M* = 5.47, *SE* = .20), *p* = .036, *d* = 0.61. With respect to covariates, results showed a marginal significant effect of self-esteem on individuals’ motivation to seek pleasant experiences, *F*(1, 91) = 3.01, *p* = .086, η_p_^2^ = .03, and a non-significant effect of extraversion, *F*(1, 91) = 0.16, *p* = .69, η_p_^2^ = .002.

### Discussion

In support of our hypothesis, results from Study 1 showed that individuals reported a lower desire for pleasant experiences following successful, but not average, performance when the task was in progress as compared to when the task was completed. Thus, individuals’ responses to success were indeed determined by the performance situation.

Interestingly, results further showed that when the task was in progress, the desire of individuals to engage in a pleasant experience following success was even lower than following average performance. One potential explanation for this finding is that individuals who had performed successfully may have “more to lose” compared to individuals who had average performance and thus, may perceive engaging in pleasant experiences as less useful for task pursuit. Moreover, one cannot exclude that engaging in something pleasant before the next task can be viewed as self-motivating, rather than interfering, following a less satisfying or unsuccessful (i.e., average) performance outcome.

However, as the present results were found in a hypothetical context and based on self-report measures, we aimed to provide further evidence for our hypothesis. Additionally, we aimed to ensure that it is in fact the pleasantness of the experience that contributed to the effect of interest. Since in the present study participants only reported the extent of their motivation to engage in an experience of pleasant valence, we cannot rule out the possibility that the effect might also occur for individuals’ motivation to engage in an experience of another valence. Thus, in Study 2, we tested our predictions in an actual performance situation. We further chose a behavioral assessment of individuals’ seeking of experiences with valences ranging from pleasant to unpleasant in an effort to expand upon findings from Study 1.

## Study 2

In Study 2, we aimed to test our hypothesis in an actual performance situation by using a behavioral measure of pleasant experience seeking. In this study, participants worked on a cognitive ability task and were provided actual feedback on their task performance, which was indicated to be either successful or average. We chose to provide feedback on participants’ real task performance as previous research suggests that success feedback which does not correspond to individuals’ actual performance may reduce individuals’ motivation to seek rewarding experiences [27, 28]. To manipulate task completion, participants were told that they either had not yet finished the task and a similar task would follow (task in progress) or that they had finished the cognitive ability task (task completed).

To evaluate the extent to which individuals seek to engage in a pleasant experience as a function of performance outcome and task completion, participants were provided descriptions of video scenes associated with pleasant valence and reported how much they would like to watch these video scenes at that particular moment. In addition to pleasant videos, we also presented video descriptions of neutral and unpleasant valence. We did so in order to rule out that the effect can be explained by participants’ motivation to engage in an activity of any emotional valence and thus to ensure that it is the pleasantness of the experience which drives the effect of interest. Thus, we did not predict any effect of task completion on motivation to watch these less pleasant video scenes.

The use of video scenes to assess pleasant experience seeking as a function of task completion was perceived as particularly powerful due to the behavior-related nature of this assessment [29, 30]. Compared to direct self-report, this assessment allowed participants to express their spontaneous desire to seek a pleasant experience without them needing to be explicitly aware of this intention. Six video descriptions were created for the present study (one video description was derived from Gendolla [31]) and pretested for whether the descriptions could indeed be differentiated by their emotional (i.e., pleasant, neutral and unpleasant) valence.

### Pretest

Of the six video descriptions created, pleasant, neutral, and unpleasant videos were each represented by two descriptions. Twenty-eight German university students (*M*_age_ = 23.97, *SD*_age_ = 1.75; 19 females) received a questionnaire presenting the video descriptions in a randomized balanced order. Participants were asked to imagine that they would watch the video and to report for each video how they expected to feel when watching it on a single-item 7-point scale (1 = *very unpleasant*, 4 = *neutral*, 7 = *very pleasant*). To test whether the videos differed on dimensions other than pleasantness, we additionally assessed how interesting, exciting and arousing participants expected each video scene to be (1 = *not at all*, 7 = *extremely*).

First, a repeated-measures univariate analysis of variance revealed a significant effect of video description on anticipated pleasantness of the video scene, *F*(5, 22) = 22.51, *p* < .001, η_p_^2^ = .84. Post-hoc tests showed that the supposed pleasant videos were indeed evaluated to be significantly more pleasant than the supposed neutral videos, *ps* < .001, *d*z ≥ 1.63, and these, in turn, more pleasant than the supposed unpleasant videos, *p*s < .001, *d*z ≥ 1.45. Means and standard deviations for perceived pleasantness of the video descriptions are displayed in Table 1. Additional repeated-measures univariate analyses of variance revealed significant effects of valence on anticipated interestingness, *F*(2,28) = 5.44, *p* = .01, η_p_^2^ = .28, and arousal, *F*(2, 28) = 24.76, *p* < .001, η_p_^2^ = .64, as well as a marginal significant effect on anticipated excitement of watching the video scene, *F*(2, 28) = 3.03, *p* = .064, η_p_^2^ = .18. Post-hoc tests showed that pleasant videos were evaluated as less interesting than unpleasant videos, *p* = .01, *d*z = 0.51, but not neutral video scenes, *p* = .19, *d*z = 0.25. Pleasant videos were further evaluated as more arousing, *p* < .001, *d*z = 1.08, and exciting, *p* = .019, *d*z = 0.46, than neutral videos, but did not significantly differ in arousal, *p* = .40, *d*z = 0.16, and excitement, *p* = .42, *d*z = 0.15, from unpleasant video scenes.

**Table 1:** Group Means and Standard Deviations of Pretest Video Scene Pleasantness Ratings (*N* = 28).

Video Scenes	*M* (*SD*)
(1) The New Year's Eve party. A documentary about four friends celebrating and dancing together.	5.41 (0.93)
(2) A wedding like in “Thousand and One Nights”. Family and friends come together and celebrate three days and nights.	5.67 (1.07)
(3) Over the course of time. A historian reflects on the demographic changes in Germany.	4.11 (0.85)
(4) From water to ice. A video documenting this physical phase transition.	4.11 (0.80)
(5) Poverty makes sick. A report about the dramatic risks of the social imbalance for health.	2.93 (0.87)
(6) The suffering of the civilian population. A report about the violation of human rights, infringements and violence during wars.	2.26 (1.10)

Together, these results show that the six video descriptions can be systematically differentiated by their valence of pleasant, neutral and unpleasant. Moreover, these results further suggest that investigating motivation to watch not only pleasant but also less pleasant videos as a function of performance outcome and task completion will help to rule out the possibility that the effects can be explained by the extent to which the video scenes are perceived as interesting, exciting and arousing.

### Method

#### Participants and Design

One hundred fifty-seven German university students participated in the experiment. A between-participants design was employed with the factors performance outcome (successful vs. average) and completion of the task (task in progress vs. completed), and the extent of pleasant experience seeking as the dependent variable. Participants were randomly assigned to one of the two task completion conditions. The assignment to either the successful or average performance outcome condition was based on participants’ actual performance on the task. For this purpose, participants’ results from the task were compared with the results of 121 undergraduate students who had performed an identical task (*M*_age_ = 21.74, *SD*_age_ = 4.33). Thirty-six participants were assigned to the successful outcome condition (in progress/completed: *n* = 17/19) and 79 participants to the average outcome condition (in progress/completed: *n* = 43/36). As outlined in “The Present Research”, our main hypothesis did not address individuals with below average performance. Thus, the data from these participants was not considered in the subsequent analyses. The final study sample included 115 participants (*M*_age_ = 21.89, *SD*_age_ = 2.50; 56 females).

#### Procedure

Participants completed the study on personal computers in a laboratory. At the beginning of the experiment, participants were informed that the study would assess their concentration ability, which was emphasized to be one of the most important predictors of students’ academic success. First, participants responded to items assessing self-esteem and extraversion. The items were embedded among filler items assessing participants’ concentration and learning skills. Participants also answered questions regarding demographic information.

Second, participants were informed that their concentration ability would be assessed in a “figure-recognition task”, with recognition tasks described to be the most frequently used method in assessing cognitive ability. The task was employed as a ‘filler’ task for participants to engage in and was not intended to measure cognitive ability in any sense other than to assign the performance outcome factor. Participants received detailed instructions, performed a trial run, and then worked on the task for 6 min. In the task, participants had to correctly estimate the number of target figures (i.e., circles) among distracting figures (i.e., triangles) displayed on the computer screen for 2 s. Following the task, participants received feedback on their performance (successful vs. average outcome). They were further informed that either they had not finished the assessment and would subsequently work a similar recognition task (task in progress) or that they had finished the entire assessment (task completed).

Participants further read that after completing the cognitive ability task (for the task completed group) or before working on the next task (for the task in progress group), they would watch a video scene. They were asked to report how much they would like to watch each of the six video scenes based on the descriptions presented. They were then asked to report how they felt after receiving their performance feedback. Emotions related to task feedback were assessed at the end of the experiment in order to conceal the purpose of rating the video descriptions. Lastly, participants were asked to report their perceptions of the intent of the experiment. Participants completed the study after approximately 40 min. Although participants were not presented with the video scenes described, they were shown landscape pictures selected from the Geneva affective picture database, which had been developed to induce positive affect [32], as compensation. They were thanked, debriefed by the experimenter, and financially rewarded for their participation.

#### Performance in the task (successful vs. average outcome conditions)

Participants were informed that their concentration ability would be assessed in terms of the number of correctly estimated trials compared to the average performance of other students in their age group (see participants and design section for details), in order to provide more insight into their concentration ability. Participants who achieved a higher performance outcome compared to other students (successful outcome) were given the following feedback: “Your concentration ability is better than the concentration ability of students in your age group as indicated by previous research. Your concentration ability is outstanding.” Participants who achieved an average performance compared to other students (average outcome) read: “Your concentration ability corresponds with the concentration ability of students in your age group as indicated by previous research. Your concentration ability is average.”

#### Manipulation of task completion (task in progress vs. completed conditions)

After receiving feedback on their performance in the task, participants in the task in progress condition read:

The assessment of your concentration ability is not yet complete. You will now proceed to the next recognition task, a letter-recognition task. The first figure-recognition task assessed your concentration ability in the visual-figural domain, whereas the second letter-recognition task will assess your concentration ability in the visual-verbal domain.

In the task completed condition, participants were thanked for finishing the figure-recognition task and informed that the assessment of their concentration ability in the study was now complete.

#### Measures. Self-esteem and extraversion

Similar to Study 1, self-esteem and extraversion were assessed by Rosenberg’s [24] 10-item Self-Esteem Scale and a 12-item extraversion scale [26]. Participants were asked to indicate the extent to which they disagree or agree with each of the statements on a 7-point scale (1 = *strongly disagree*; 7 = *strongly agree*; α = .80 for self-esteem, α = .73 for extraversion).

#### Pleasant experience seeking

Six descriptions of video scenes as outlined in the pretest and Table 1 were presented in a randomized order. Participants were informed that from these videos they would watch one video scene for about 2 min and that the scene would be chosen depending on their ratings of the descriptions. Participants then rated how much they would like to watch each of the video scenes at that moment (1 = *not at all*; 7 = *very much*). Ratings across each of the video scenes of the same valence category (pleasant, neutral and unpleasant) were then averaged.

#### Positive emotions about the outcome

To test whether participants who had a successful outcome experienced stronger positive emotions than those who had an average outcome, we assessed positive emotions in terms of four items (“happiness,” “enjoyment,” “pride,” “relief”) derived and translated from the Geneva Emotion Wheel [33, 34]. Participants were asked to report the extent to which they had experienced each of these emotions when they received their performance feedback on a 7-point scale (1 = *not at all*; 7 = *extremely*). Average scores were used as composites of positive emotions (α = .91).

### Results

#### Positive Emotions about the Outcome

We conducted a univariate analysis of variance on positive emotions with performance outcome and task completion as between-participants factors. Results showed a significant effect of outcome, *F*(1, 106) = 53.77, *p* < .001, η_p_^2^ = .34. The effects of task completion and the interaction between task completion and outcome on the experience of positive emotions were not significant, *F*s ≥ 1.21, *p*s ≥ .27, η_p_^2^ ≤ .01. Participants with a successful outcome reported a significantly stronger experience of positive emotions (*M* = 4.88, *SD* = 1.40) than participants with an average outcome (*M* = 2.92, *SD* = 1.26). These results suggest that feedback indicating above average performance in the task led to stronger positive emotions than feedback indicating average performance, both when the task was completed and in progress.

#### Pleasant Experience Seeking

We first conducted a multivariate analysis of variance on individuals’ motivation to watch video scenes, including performance outcome and task completion as the between-participants factors and valence of the video scenes as the within-participants factor. Self-esteem and extraversion were included as covariates in the analysis. Results of Levene tests showed that the assumption of variance homogeneity was not violated by unequal cell sizes, *p* = .61, and allow for a full interpretation of the present results [35]. Results showed non-significant effects of performance outcome, *F*(2, 102) = 0.81, *p* = .45, η_p_^2^ = .02, and task completion, *F*(2, 102) = 2.10, *p* = .13, η_p_^2^ = .04. The effect of the valence of video scenes on individuals’ motivation to watch the video scenes was significant, *F*(2, 102) = 5.24, *p* = .007, η_p_^2^ = .09, as was the interaction between outcome, task completion and valence, *F*(2, 102) = 4.10, *p* = .019, η_p_^2^ = .07.

In a next step, we conducted univariate analyses of variance in order to test the effects of performance outcome and task completion on motivation to watch pleasant, neutral or unpleasant video scenes separately. With respect to motivation to watch pleasant video scenes, results showed a non-significant effect of performance outcome, *F*(1, 103) = 1.55, *p* = .22, η_p_^2^ = .02, and a significant effect of task completion, *F*(1, 103) = 5.69, *p* = .019, η_p_^2^ = .05. As expected, this effect was qualified by a significant effect of the interaction between outcome and task completion, *F*(1, 103) = 5.17, *p* = .025, η_p_^2^ = .05. With respect to motivation to watch neutral and unpleasant video scenes, results of univariate analyses of variance did not show significant effects of the outcome, task completion, or the interactions between outcome and task completion, *F*s ≤ 2.01, *p*s ≥ .16, η_p_^2^ ≤ .01. These results show that, indeed, only motivation to engage in a pleasant experience varied as a function of performance outcome and task completion.

**Fig 2:**
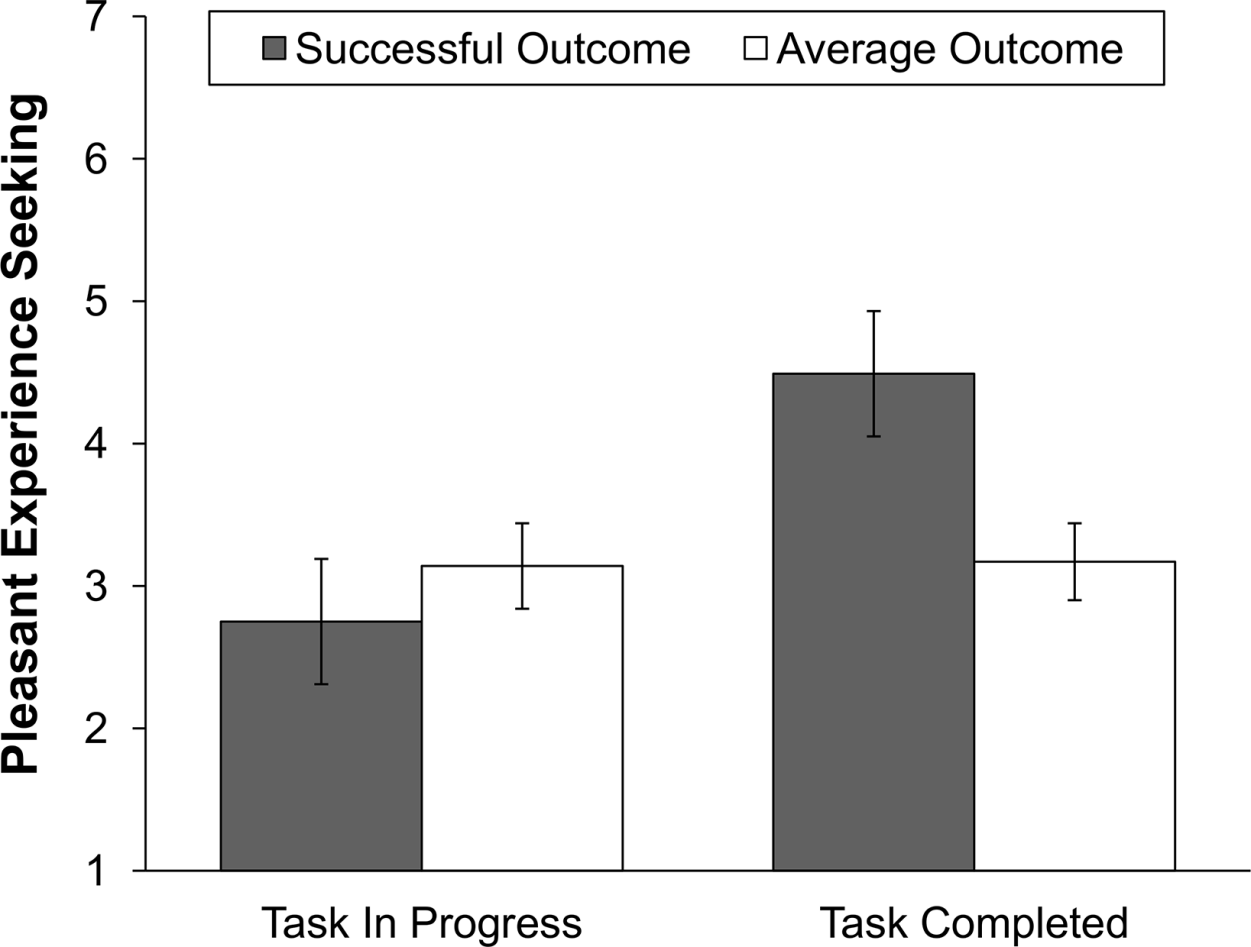
Group means and standard errors for motivation to seek pleasant experiences (i.e., watch pleasant video scenes) after achieving a successful versus an average outcome in both the task in progress and task completed conditions.

We conducted post-hoc tests to investigate the differences in motivation to watch pleasant video scenes across the performance outcome and task completion conditions in detail. Means and standard errors for participants’ motivation to watch pleasant videos across the conditions are displayed in Fig 2. In line with our hypothesis, results showed that participants who achieved a successful outcome reported significantly lower motivation to watch pleasant videos when the task was in progress (*M* = 2.75, *SE* = .44) than when the task was complete (*M* = 4.49, *SE* = .44), *p* = .007, *d* = 0.98. No significant difference across the task completion conditions in motivation to watch pleasant videos was found for participants who received average performance feedback, *p* = .93, *d* = 0.02, indicating that the effect of task completion was indeed specific to successful participants.

In addition, results of post-hoc tests further showed that participants who achieved a successful outcome reported significantly higher ratings for their motivation to watch pleasant videos (*M* = 4.49, *SE* = .44) compared to participants with average performance (*M* = 3.17, *SE* = .27) in the task completed condition, *p* = .013, *d* = 0.74. However, there was no significant difference in motivation to watch pleasant videos between participants with successful and average outcomes in the task in progress condition, *p* = .47, *d* = 0.21. With respect to covariates, results showed a significant effect of extraversion on individuals’ motivation to watch pleasant videos, *F*(1, 103) = 5.63, *p* = .02, η_p_^2^ = .05, and a non-significant effect of self-esteem, *F*(1, 103) = 0.004, *p* = .95, η_p_^2^ < .001.

#### Supplemental Analyses

Given the results showing that the effect of task completion had a significant impact on individuals’ motivation to watch pleasant (but not neutral or unpleasant) videos following a successful outcome in the task, as predicted, we additionally inspected the extent of individuals’ motivation to watch pleasant videos *compared* to less pleasant videos. Results of the analysis of variance including task completion as the between-participants factor and valence of the video scenes as the within-participants factor did not show a significant effect of task completion or valence on the motivation to watch video scenes, *F*s ≥ 2.12, *p*s ≥ .14, η_p_^2^ ≤ .14. The effect of the interaction between task completion and valence, however, was significant, *F*(2, 27) = 5.52, *p* = .01, η_p_^2^ = .29. Post-hoc tests showed that in the task completed condition, participants’ motivation to watch pleasant videos after a successful outcome was significantly higher than their motivation to watch unpleasant videos (*M*_*pleasant*_ = 4.43 vs. *M*_*unpleasant*_ = 3.12), *p* = .02, *d*_*z*_ = 0.47, and did not significantly differ from their motivation to watch neutral videos (vs. *M*_*neutral*_ = 4.36), *p* = .88, *d*_*z*_ = 0.03. In the task in-progress condition, participants’ motivation to watch pleasant videos was lower by trend compared to their motivation to watch unpleasant video scenes (*M*_*pleasant*_ = 2.70 vs. *M*_*unpleasant*_ = 3.73), *p* = .062, *d*_*z*_ = 0.37, and was significantly lower compared to their motivation to watch neutral video scenes (vs. *M*_*neutral*_ = 4.55), *p* < .001, *d*_*z*_ = 0.75. There were no significant effects of the covariates extraversion and self-esteem on the motivation to watch video scenes, *F*s ≥ 0.004, *p*s ≥ .95, η_p_^2^ < .001.

### Discussion

Results from Study 2 provide support for our hypothesis and replicate the results from Study 1 in a real performance situation by using a behavioral assessment of motivation to seek pleasant experiences. More specifically, the present results showed that after attaining successful performance, but not average performance, individuals reported a lower desire to engage in a pleasant experience when the task was in progress as compared to when the task was completed. In fact, when another task was lying ahead, successful performance did not lead to greater pleasant experience seeking than average performance. In other words and similar to the results of Study 1, when the task was not yet complete, individuals seemed not to benefit from their successful performance, at least from a hedonic perspective of engaging in pleasant experiences.

Importantly, results of Study 2 ruled out an alternative explanation of the results of Study 1 by showing that it is specifically the motivation to engage in experiences of pleasant (and not neutral or unpleasant) valence that varies as a function of performance outcome and task completion. Moreover, supplemental analyses of individuals’ preferences for the experiences of different valence could provide additional insight and support for our prediction. Results showed that individuals sought pleasant experiences more following successful performance than unpleasant experiences when the task was complete. However, when the task was in progress, individuals tended to prefer unpleasant experiences more than pleasant experiences. Because these findings are based on intraindividual comparisons and should be interpreted with caution due to differences between the video scenes, future investigations are of interest in exploring whether and why individuals may “dampen” their success experience when the task is not yet complete.

## General Discussion

Previous research suggests that seeking pleasant experiences following success may help individuals to enjoy their performance and to meet their hedonic needs [4–6]. In the present research, we proposed that individuals will seek pleasant experiences following successful performance depending on the situation in which this success has been achieved. Results of two studies provided support for our prediction by showing that individuals expressed a lower desire to engage in pleasant experiences following successful performance when the task was still in progress as compared to when the task was completed. These results were found in a first study using a hypothetical context in which both performance outcome and task completion were manipulated, using a self-report measure of pleasant experience seeking. In a second study, we were able to replicate the findings in an actual performance situation using a behavioral assessment of pleasant experience seeking. Whereas results of Study 1 unambiguously showed that the effects were not driven by any interpersonal difference of participants, results of Study 2 showed that the effects also hold true in an actual performance situation and are specific for motivation to engage in experiences of pleasant valence. Together these findings provide clear evidence that the performance situation determines how individuals respond to their successes and whether they are likely to engage in gratifying and rewarding experiences. Moreover, the present results further suggest that when the task is still in progress, successful performance does not lead to a stronger desire for pleasant experiences compared to average performance (Study 2) and even seemed to reduce this hedonic motivation (Study 1).

These findings are in line with previous research showing that pleasant experiences signal that the task is finished and may interfere with individuals’ focus on the task [12, 16] as well as research suggesting that individuals forego immediate pleasures for the sake of future benefits [17–19]. In other words, one might assume that engaging in pleasant experiences following successful performance may indeed indicate that one’s goal has been successfully reached when the task is finished, but not when the task is still in progress―in these situations, rather than seeking pleasure from one’s present success, individuals might want to seek pleasure from future outcomes or a successful goal pursuit. Future studies are important in investigating the underlying mechanisms of the effect of task completion on seeking pleasant experiences as well as the potential beneficial consequences of the tendency to forego pleasant experiences with respect to individuals’ persistence or motivation.

Importantly, future research is also needed to investigate the personal consequences of the tendency to forego pleasant experiences following one’s successes. More specifically, doing something enjoyable directly following success not only reflects the desire to give into temptation but also the desire to savor and to enjoy one’s successful performance. Previous research has underscored the notion that enjoying and savoring one’s positive events is highly important for individuals by contributing to life-satisfaction [3, 7]. As such, one might also assume that a continuous foregoing of pleasant experiences following successes for the sake of future tasks might have also negative effects, for instance, on individuals’ long-term happiness. In light of the prevalence of situations in which one’s duties or tasks are succeeded by another in daily life, consideration of these effects in future research is particularly relevant.

Thus, the present research provides new empirical evidence showing that individuals are less motivated to seek pleasant experiences following success in a task when the task is not yet complete as compared to complete, and proposes important implications with respect to both the benefits and costs of foregoing such pleasant experiences.

## Supporting Information

S1 Dataset This zipped folder file contains the SPSS datasets of Study 1 (Study 1_dataset.sav), pretest of Study 2 (Study 2_dataset_pretest.sav) and Study 2 (Study 2_dataset.sav). (ZIP)
